# Meal Plan in the Treatment of Anorexia Nervosa: A Way of Feeding the Disorder and Starving the Patient

**DOI:** 10.5539/gjhs.v5n1p112

**Published:** 2012-11-11

**Authors:** Maria João Padrão, Maria Raquel Barbosa, Joaquim Luís Coimbra

**Affiliations:** 1 Faculty of Psychology and Education, University of Porto, Porto, Portugal

**Keywords:** anorexia nervosa, meal plan, control, traditional medical model, critical perspective

## Abstract

For the mainstream Psychology/Psychiatry, anorexia nervosa is considered an eating disorder characterized by the low body weight and by the restrictive eating pattern. The traditional psychiatric treatment consists in the establishment of a meal plan that must be scrupulously followed and, most frequently, in pharmacological treatment. We propose an alternative conceptualization of anorexia nervosa that envisages this disorder as pertaining to the control domain. In this sense, we formulate psychiatric intervention as a “pact with anorexia”, once it follows the very same logic, prohibitions and self-impositions of the disorder. Specifically, we envisage the meal plan as a way of maintaining anorexia, instead of suppressing it. As we could observe in our four year research project, in which we’ve followed several anorectic female patients, those who were more committed to their psychiatric treatment were precisely those who had more difficulty in recovering from anorexia – i.e., from renouncing the control from which the disorder lives. Finally, we suggest some fundamental underpinnings to an effective therapeutic approach, based in our conceptualization and understanding of the disorder.

## 1. The Usual Way of Conceiving Anorexia Nervosa

For the mainstream Psychology/Psychiatry, anorexia nervosa is considered an eating disorder characterized, firstly, by the low body weight and by the disordered, restrictive eating pattern. Although conceptions and ideas about anorexia nervosa have clearly suffered many alterations over time, in mainstream clinical research and practice very little has changed. The notion of anorexia as an illness remains basically untouched and unchallenged. Symptoms are categorized into syndromes that are operationally defined and objectively analysed. Since anorexia nervosa was conceptualized as a psychiatric disorder and included in the Diagnostic and Statistical Manual of Mental Disorders (DSM) (critically recognizing this classification as the expression of the trends of reification and naturalization of Psychology/Psychiatry, ideological mechanisms that, according to Horkheimer and Adorno (1947; published in English 1972), both in collective/societal level and in individual/psychological level have functions of protection and legitimation of political interests), the diagnostic criteria for anorexia nervosa are focused on the physical body and in perception distortions and biomedical factors that can be observed and measured in an objective manner.

The DSM criteria (DSM-IV-TR) for anorexia nervosa include: a refusal to maintain body weight at or above a minimally normal weight for age and height; an intense fear of gaining weight; a disturbance in the way in which one’s body weight or shape is experienced, undue influence of body shape on self-evaluation, or denial of the seriousness of the current low body weight; and amenorrhea in postmenarcheal females (APA, 2000).

Anorectics are considered psychologically *disordered* and are analyzed, in terms of comorbidity, in several psychological dimensions that promote or are caused by anorexia, such as depression, anxiety, alexithymia, obsessive-compulsive behavior or borderline personality disorder. Anorexia nervosa is associated with a distorted self-image which may be maintained by various cognitive biases that alter how the affected individual evaluates and thinks about her or his body, food and eating.

The line this paradigm makes between illness and non-illness is, hence, very clear.

Although several research projects over the years have tried to understand this disorder and diverse theoretical approaches have tried to build conceptual and causal models to its comprehension, the etiology of anorexia nervosa remains unknown. Independently of the conceptions about anorexia and their evolutions, anorexia remains globally unexplained and considered an *eating disorder*, fact that is responsible for many myths and misconceptions about the anorectic lived experience, which, in turns, inhibits the progresses on the intervention level. Therapeutic interventions within mainstream practice remain held to notions such as “eating”, “disorder”, “objective body”, “body mass index”, “distorted self-image” or “cognitive bias”. This model of intervention, as we’ll analyse later, seems to promote and confirm anorexia nervosa, imprisoning the woman and her existence in the colonization of the disorder.

In this sense, we can say that contextualizinganorexia nervosa within the boundaries of clinical practice and objectifying the person and the body promotes a broken and fracturent dialogue between nature and society, pathology and normality, clinician and patient, between anorexia nervosa as an illness and anorexia nervosa as a human experience (Sanz & Burkitt, 2001). From a phenomenological standpoint, it’s not viable to work with notions such as pathological body or distorted perception. Rather, lived experience of the woman with anorexia nervosa^1^ must be considered in the context of her lived reality and worked with in a dialogical manner – which implies that the therapist must give up his objective perspective to encounter phenomenologically the anorectics´ meanings.

Our conceptualization of anorexia nervosa is quite different from the usual one and, thus, our therapeutic approach is, accordingly, divergent from the mainstream model.

## 2. An Alternative Conceptualization of Anorexia Nervosa

Our observations and reflections about anorexia nervosa derive from a four year research project, in which we’ve followed several female patients diagnosed with anorexia nervosa, restrictive type (age range: 15-56 years; body mass index (BMI) range: 11-16; illness duration range: 2-36 years; psychiatric accompaniment range: 1-31 years) in the context of group psychological interventions. We’ve conducted psychotherapeutic interventions with several groups of patients, with a six-months duration, on a weekly basis. Our therapeutic approach is based on a constructivist, developmental and phenomenological standpoint and integrates the body as an important element of the intervention, through body-oriented techniques (stemming from dance-movement therapy, for example) and through the symbolization and embodiment of feelings and meanings by means of dance, expression and movement. We’ve adopted a pre post-assessment design in order to evaluate the interventions. The level of analysis was the individual, and the criteria of weight, BMI and relation to the body/bodily lived experience were considered.

In the tables bellow are presented the participants of the main study and their evolution in terms of weight and BMI (at the time of the pre-intervention, post-intervention and follow-up) in order to obtain a general perspective of the results of our intervention.

**Table 1:** Description of the participants of the main study (pre-assessment)

Name	Age (years)	Weight (Kg)/BMI	Disorder’s length (years)	Psychiatric accompaniment (years)
Sara	23	37,6/14,2	6	4,5
Isabel	16	43,8/16,2	2	2
Luísa	26	40,8/15,7	12	11
Ana	19	31,6/12,3	3	3
Teresa	37	41,7/14,7	4	3
Maria	30	31,6/13,1	17	13

**Table 2:** Weight and BMI evolution in the participants of the main-study

Name	Weight (Kg)/BMI Pre-assessment	Weight (Kg)/BMI Post-assessment	Weight (Kg)/BMI Follow-up
Sara	37,6/14,2	50/18,8	54/20,3
Isabel	43,8/16,2	56/19,3	57,6/19,9
Luísa	40,8/15,7	45/17,3	52,6/20,9
Ana	31,6/12,3	36,6/14,3	37,8/14,7
Teresa	41,7/14,7	46,2/16,4	46,4/16,4
Maria	31,6/13,1	30,8/12,8	------------*

* We did not obtain any data regarding Maria during the follow-up stage as she did not show up for the session. She stopped being followed by the Psychiatric Service of the Hospital by her own choice. At the moment of the follow-up, she had been admitted into a private clinic.

The close attachment relationships established with the patients throughout group sessions and individual interviews, the thorough analysis of their life stories and the systematic follow-up of their evolutions, allowed us to elaborate a plausible theory of the development and onset of anorexia nervosa.

### 2.1 The Development and the Onset of Anorexia Nervosa: A Plausible Explanation

Through the comprehensive analysis of the different cases that we’ve followed, it was possible to verify that there seems to exist two important factors in the etiology of anorexia nervosa in those women:

- An enmeshed relationship with the primary attachment figure (as it was preconized by Minuchin and colleagues – vd. Minuchin, 1974; Minuchin, Baker, Rosman, Milman, & Todd, 1975; Minuchin, Rosman, & Baker, 1978);
- A pervasive feeling of uncontrollability in the onset phase of the disorder, stemming from a particular life event (for example, a context-related change or, more frequently, a modification at the attachment relationships level) or derived from an accumulation of factors capable of accentuating the necessity of control, as we know, structurant of psychological stability.

For example, for Sara (23 years old, anorectic since 17), her mother was the attachment figure with whom she established an enmeshed relationship and from whom she had an accentuated difficulty on the individuation process. The birth of her baby brother constituted the event that affected her feeling of controllability, including the relationship with the mother level. Her anorexia started there.

Ana (19 years old; anorectic since 16) had an enmeshed relationship with the father that she idealized and idolized. The school change and the concomitant frustration for not having succeed in moving to a school next to her parents’ home, forcing her to live with her grandparents, as well as the perception of the existence of conflict between the parents (which constituted a major disappointment) and the consequent (painful) grieve of the idealized *imagos* of her parents (particularly of her father) constituted the motivating factors of the feeling of uncontrollability in relation to her life and to the ideal world constructed by Ana.

Isabel (16 years old, anorectic since 14), spent all her time with her mother – her enmeshed attachment figure –, with whom she shared everything. The move to a foreign country – with all the changes and adaptations it implies – was the stressing event that disturbed her feeling of controllability and comprehension of the world.

Teresa (37 years old, anorectic since 33) had an enmeshed relationship with her six year-old son, having an excessive separation anxiety from him. She seemed to have difficulty in allowing her son’s individuation and differentiation, projecting on him her own individuation problems. The beginning of the disorder seems to be associated to a pervasive feeling of uncontrollability for which several factors seemed to have contributed: a rare disease diagnosed to her son (when he was 3 years old) and her feeling of incapacity in relation to his recovery; the deterioration of her marriage and the domestic violence from which she started to be victim of and, later, the divorce (even when desired, divorce constitutes a life change that needs adaptation). Finally, another important aspect to consider is the fact that Teresa suffered, in her childhood, a continued sexual abuse by a friend of her parents that seemed to have left in her body, in a latent manner, a feeling of uncontrollability in relation to her major self-property: her own body.

For Maria (30 years old; anorectic since 13), her main attachment figure, with whom she maintained (and kept maintaining) an enmeshed relationship was her mother (as we will analyze later, Maria didn’t recover from her anorexia). Her mother was, practically, the only person with whom she related and from whom she was absolutely dependent. The unbearable disappointment she had suffered with her father, when she was 13 years old, when she found out that he had an extramarital relationship constituted the trigger factor for her feeling of uncontrollability – until then, her father was considered by Maria as morally perfect and a good husband and father. The finding of his infidelity ruined her fundamental underpinnings of the conceptions of integrity, stability and foresee ability. This fact is agreeing with the disappointment experience in the onset of eating disorders preconized by Guidano and Liotti (1983). Additionally, she was teased at school because she was a little over weighted. Putting these two elements together, the necessity of control escalates to an extreme level. Maria was one of the serious cases of anorexia ever met by us.

In this sense, our study seems to corroborate theories that formulate anorexia nervosa as an individuation disorder (e.g. Goodsit, 1985; Guidano, 1987; Guidano & Liotti, 1983; Minuchin, 1974; Minuchin, Rosman, & Baker, 1978). We’ve verified three different dimensions that seem to support this conception. First, the referred enmeshed relationship within which the anorectic is incapable of functioning separately. The second dimension that indicates an arrest in the individuation process is the idealization of childhood. In fact, this period is considered, by these patients, as the “golden age”, where everything was perfect. The patients seem totally incapable of recognizing imperfections or tensions in their childhoods, the parents are described as ideal as well as the familial dynamics and relationships. This idealization of childhood appears as a significant deflection of their maturity and individuation process and gives us some important clues to the comprehension of the disorder. If we attend to the dynamics of the quotidian and the attachment relationships of these patients, we realize that they seem to remain children, totally dependent of the care of the attachment figure. The attachment figure – generally, the mother – is always diligent to her fragile daughter, looking out for her health, transportation and even for her alimentation – that is, the mother keeps looking out for her “child”. In this sense, we can formulate the strengthening and maintenance of an enmeshed relationship with the primary attachment figure as a “secondary gain” of anorexia, and we can comprehend this situation as a regression or a maintenance of the child *status-quo*: one who is taken care of, that doesn’t have to assume the responsibilities and the risks of an adult identity and an adult life (nor those of an adult body), that is completed within the relationship with the primary attachment figure, without any necessity of relating to *others* that could, possibly, deceiveher.

Their behavior is generally characterized by an accentuated immaturity, an excessive self-focalization and very low autonomy.

We can, thus, realize that we are in the presence of a paradox: these women search for individuation through the most basic and primitive means that they have – their own bodies – but, simultaneously, desautonomized themselves and return to (or remain at) the *status quo* of children. Here inhabits the profound contradiction of the anorectic lived experience that must be recognized and worked through to an effective therapeutic evolution.

Finally, the third area that indicates us a deep difficulty on the individuation level is sexuality. These women invariably express an avoidance of an adult sexual identity. However, what we’ve realized in our research project is that is seems to be more an ambivalent relationship with femininity and sexuality (as shown by the desire and avoidance of feminine curves and feminine and sensual movements) than a real avoidance of these dimensions (Padrão & Coimbra, 2011) – what appears to be in the line of the paradox above presented.

Within our group interventions and with the use of the body, we were able to verify paradoxical and ambivalent feelings (desire vs avoidance) related to sensual movement and femininity – for example, when a sensual movement was proposed, some patients reacted with a refusal to perform it, although curiosity was very high, while others, at the same time they were excited when they saw the movement and revealed willingness to do it, immediately started crying once they started to perform it (ibid.).

Sara (23 years old, anorectic since 17) reacted violently to our first proposals to express femininity and sensuality, insulting the therapist and leaving, aggressively and in tears, the session. Later, in the following session, she referred she was ashamed of her behavior and that she didn´t realize why she had reacted that way.

The ambivalent relationship with femininity and sexuality could also be seen in comments such as: ‘‘Wow! That kind of movements really looks good on you! I find it very sensual and beautiful. I’m just not ready to do it. I guess I’ll never be ready…’’ (Manuela, 56 years old; anorectic since 20). ‘‘I like to watch that movement but I don’t want to do it. That’s all’’ (Mafalda, 24 years old; anorectic since 17). ‘‘I don’t know what I think. On the one hand, I like it, but on the other…I don’t know…I guess I’ll be just watching for today’’ (Rita, 38 years old; anorectic since 34).

Sexuality is known to be an area profoundly impaired in anorexia nervosa (cf. Ghizzani & Montomoli, 2000; Morgan, Wiederman, & Pryor, 1995; Tuiten, Panhusysen, Everaerd, & Koppeschaar, 1993; Widerman, Pryor, & Morgan, 1996). For example, Widerman et al. (1996) showed that self-ratings of current sexual satisfaction, as well as masturbation experience, were inversely related to the degree of restriction of caloric intake among women with anorexia nervosa, and revealed that these women exhibited less sexual interest and more negative affect during sex than a normative sample (Morgan et al., 1995). Generally, difficulties in sexual functioning are associated by researchers to endocrine and neuroendocrine alterations, supporting the hypothesis that the problems in these patients’ sexual life arise only after the emergence of hypogonadism, as a consequence of emaciation. According to Tuiten et al. (1993), the hormonal and associated psychosexual changes are central to the pathogenesis of anorexia nervosa.

The ambivalence we found in what concerns sexuality and femininity, although supporting the studies that demonstrate a difficult relationship with sexuality in patients with anorexia nervosa, seems to stress the central role psychological factors might play in this dimension of the problem. What our results seem to reveal is that sexuality is avoided in the same amount that it is desired and therefore cannot be attributed exclusively to an endocrine alteration. Perhaps psychological factors, such as assuming individuality, independence, and adulthood may play a central role not only in terms of sexuality but with respect to the onset and maintenance of the disorder (Padrão & Coimbra, 2011). Most scientists believe that the altered balance of ovarian steroids and central nervous system neurotransmitters might explain the lack of sexual interest. In fact, endocrine and neuroendocrine alterations can be found in depressed patients and are associated with loss of sex drive (vd. Angst, 1998; Hayes et al., 2008; Hirschfeld & Robert, 1998; Matthew & Weinman, 1982). Moreover, according to some reports, these alterations seem to precede malnutrition, which supports the hypothesis that difficulties in sexual functioning observed within anorectic patients have an origin as complex as that of the eating disorders (Ghizzani & Montomoli, 2000).

Throughout the group interventions, this ambivalence was obvious and its consciencialization and experience through the body and through the movement were crucial to its resolution. The resolution of this ambivalence was transformative of these women, both in the relation to the body level (that was also evident in an accentuated change in the adopted look that turned out to be more feminine and attractive) and in the individuation level – and consequently, in their capability of establishing a romantic, intimate relationship.

At the end of the intervention, Sara (23 years old, anorectic since 17) started a relationship with a boy with whom she fell in love and was capable of recognizing that her strong avoidance of sexuality was due to fears of becoming an adult.

### 2.2 Controlaboveall

The obsession for control – that is recognized as a central dimension in anorexia nervosa (e.g. Bruch 1962 & 1973) – is, in fact, a transversal characteristic to all existential dimensions of these women. In their quotidian, control is systematically experienced in food intake, which is exhaustively counted to the minimal gram, as well as in the obsessive monitorization of their body weight and of the most subtle corporal contours (where they can always find fat).

What seems important to notice is that the loss of weight had functioned, in every patient’s life – in the specific phase each one of them was living, characterized by uncontrollability (as we’ve described above) – as a re-establishment of the feelings of control and power. Every single patient described the feeling of power and efficacy whenever the weight became lower and lower. Their preoccupations were, at a conscious level, totally focused on losing weight. Thus, in their quotidian, control turned out to be experienced as a survival condition and, consequently, systematically promoted and exerted in all levels: in food intake (counted to grams and calories), in the weight and body fat monitorization, in the compulsive and exhaustive readings about ways of losing weight, food and health (in which they could “discover” more and more harmful aliments that had to be avoided from entering their bodies), in the hypervigilance in relation to others’ reactions to themselves or in the constant alert state that incapacitated them to sleep (sleep disorders are a common feature in anorexia – vd. Beumont, 1995; Kim et al., 2010).

In the therapeutic evolution of these patients, the fears of “losing control” and “growing fat without stopping” are common and surpassing them implicates the re-establishment of trust in their own bodies and in the readings they make of their bodily sensations. In fact, interoceptive awareness (the ability to discriminate between individual sensations and to respond accurately to emotional states – vd. Garner, 1984) and interoceptive sensitivity (the extent of an individual’s sensitivity to bodily signals) seem to be profoundly impaired in anorexia nervosa (Fassino, Pierò, Gramaglia, & Abbate-Daga, 2004; Garner et al., 1983; Lilenfeld, Wonderlich, Riso, Crosby, & Mitchell, 2006; Matsumoto et al., 2006; Pollatos et al., 2008).

This necessity for control is also explicit at the body movement level. Within group sessions, we could observe that control and hypervigilance were so extreme that incapacitated them for performing danced movement – i.e., expressive movement guided by the music. The rhythm of the movement was totally imposed by these women, being the music completely indifferent to their body movement (that was reduced to the minimum possible). Following the music rhythm would imply escaping from total control because something exterior was commanding their most valuable means of survival: body control. These women were also incapable of relaxing, because that would imply a decline of hypervigilance and a focalization in bodily sensations (which, as we can comprehend, for these women, are seriously threating). At a conscious level, body was highly invested by these women, who wouldn’t allow themselves to pollute it with poisoned food; at a tacit level, they treated their bodies as enemies that betrayed them and should, thus, be silenced and treated with suspicion. This distrust of the enemy was compensated by an overdependence on external parameters, definers of personal value: weight, body mass index, body fat percentage, clothing size, etc. – that is, the numbers that matter for anorexia nervosa.

### 2.3 Anorexia Nervosa as a Disorder Pertaining to the Control Domain

Our thesis about anorexia nervosa is that it is not a disorder associated to a body image disturbance or to the persecution of determined socio-specific beauty ideals associated to thinness. The beauty ideal of a “thin woman” has been considered, especially by the critical approaches of the feminist paradigm, as a primary factor in the onset and propagation of eating disorders in contemporary societies (vd. Chernin, 1983 & 1986; Fallon et al., 1994; Lawrence, 1984; Orbach, 1993). As this research project has shown us, the persecution of beauty ideals vehicled by the media doesn’t seem to be the cause or the social explanation for anorexia nervosa. On the contrary, there actually seems to exist a corporal avoidance of beauty, femininity and attractiveness in these women.

Anorexia nervosa is, in our perspective, an embodiment of the necessity of control, repeatedly frustrated throughout the individual’s existence, accentuated by the conjuncture of contemporary western societies, characterized by individualisation (Bauman, 2001; Lasch, 1979; Lipovetsky, 1983; Lipovetsky & Charles, 2004; Martuccelli, 2002; Twenge, 2006), disindividuation (Stiegler, 2004), “control” (Deleuze, 1990), invisibility (Innerarity, 2004), virtuality, visibility, symbolic deficit (Coimbra & Menezes, 2009; Stiegler, 2004 & 2005), absence of a meaning to life, hyperconsumerism, paradox and contradiction (Lipovetsky& Charles, 2004) which, for several reasons that escape from the scope of this article, seem to promote a greater feeling of uncontrollability in young women, compared with men – although the desindividuation tendency, obviously causative of the feeling of uncontrollability, is transversal to gender.

As Zizek (1999) points out, in contemporary risk society, the choice is absolutely “free”; however, the freedom to decide that the modern subject possesses is not the freedom of someone who can freely decide his own destiny, but the “freedom source of anxiety” that compels him to constantly assume choices without any knowledge of their consequences. Anorexia nervosa solves the “freedom source of anxiety” through the self-imposition of rigidly defined rules and limits towards the accomplishment of short-term objectives, remaining the long-term ones (i.e., “life projects”) romanticized and idealized and, for that reason, detached from present decisions. To the sensation of uncontrollability and undefinition provided by the current society’s conjuncture, anorexia nervosa opposes the extreme control of the most elementary need of human being – alimentation.

In this context – which conjugates collective disindividuation with factors like the feeling of uncontrollability and undefinition, the difficulty of finding a sense and a meaning for personal existence and the generalized symbolic deficit – as Lipovetsky (2006) notes, hyperconsumption is seen as a way of fighting against the natural fatality of life, as an “anti-destiny” and as an attempt of finding a personal meaning to existence – the hyperconsumer individual is someone searching for himself (ibid.).

The anorectic is a vulnerable, fragile and unprotected hyperconsumerist of the desindividuation and preventive tendencies of contemporary risk societies, in the sense Lipovetsky (2006) conceptualizes hyperconsumerism.

We can, thus, conclude that contemporary society contributes to the validation and potentiation of two central dimensions in the etiology and phenomenological lived experience of anorexia nervosa: the surrenderness to the concrete (promoted by the symbolic deficit and by the assimilation of the incalculable by the calculable) and the pervasive feeling of uncontrollability.

It’s in this sense that we can conceptualize anorexia as symbolizing an extreme obedience to contemporary demands characterized by contradiction and paradox. Anorexia constitutes both a symptom of a social psychopathology and a resistance force to that same psychopathology. Anorexia seems to constitute a voice of resistance to the individuation incapacity as well as an evident symptom of the prevalent insanity of contemporary societies, obsessed with control at all levels. In fact, individual psychopathology of theses suffering singularities can be seen as a glaring alert of the inadequation and insanity of the structurant underpinnings of the psychological of hyperconsumerist societies.

Additionally, there is another central dimension of anorexia to which contemporary western conjuncture contributes highly. Contemporary risk society constructs, unconsciously, higher risks than those against which it tries to prevent – one of them is the loss of autonomy. As Elias (1987/1991) had already referred, the passage to the adult world is more and more interdicted and is later and later. The “preparation” to “be an adult” takes several years and, during that preparation phase, the juvenile is totally dependent on his parents. In fact, juveniles remain children until a late age and, until they arrive to the “adult world”, they are totally dependent on their parents who have the (prolonged) function of providing them the necessary emotional and financial security and trust so that they can arrive to adulthood fulfilled and victorious.

As we have already referred, the autonomy of our women was profoundly affected. In fact, despite the idiosyncratic familial and relational dynamics in which each woman had developed and that promoted the overdependence of the caretakers and the emotional and functional immaturity, there is a broad social conjuncture that, more than just accepting, endorses that overdependence.

## 3. Traditional Medical Model of Intervention as a Pact with the Disorder

### 3.1 The Medical Model of Intervention

The traditional psychiatric treatment of anorexia nervosa consists in the establishment of a meal plan that should be scrupulously followed – the patient isn’t allowed to eat less or (even) more than what is prescribed. Simultaneously, in the great majority of the cases, patients are given pharmacological treatment to deal with the concomitant problems of depression, anxiety or, within the medical standpoint, what is seen as psychotic symptoms (like body image disturbance). The pharmacological measures of psychiatry (the *toxicomania*, using the term of Stiegler (2006)) preconize the chemical control of emotions and, essentially, of human suffering. Generally, patients are medicated with antidepressants (to deal with depressive symptoms, although the results that have been observed in anorexia nervosa are disappointing - vd. Bacaltdurk & Kaio, 2005; Claudino, 2005; Fernandes, 2007; Jimmerson, Wolfe, Brotman, & Metzger, 1996; Kaye, Nagata, Weltzin, Hsu, & Bulik, 1991), anxiolytics (to the relief of anxiety), hypnotics (for the frequent sleep disturbances) and, finally, antipsychotics. Antipsychotics are often prescribed in resistant and agitated patients, with severe body image disturbance (Bacaltdurk & Kaio, 2005; Claudino, 2005; Fernandes, 2007; Jimmerson et al., 1996; Kaye et al., 1991).

We can inquire about the meaning that underlies the prescription of antipsychotics. From a phenomenological and constructivist point of view, we can’t say that the woman with anorexia nervosa has a distorted perception (because disordered or ill); additionally, resistance should be seen as a defense mechanism of the systemic coherence and of the cognitive organization instead of a factor to eliminate, without signify, withdrawing, this way, the patient’s autonomy in his/her therapeutic and developmental process.

Additionally, psychiatric sessions constitute, for anorectic patients, a routine for weight control, where reflections about the causes of their tense relationship to body and food are treated as matters of secondary importance.

### 3.2 The Meal Plan “Feeds” Control and Not the Patient

Given the centrality of control in the etiology and in the lived experience of anorexia, as we’ve presented above, we would like to propose a reflection about the simple prescription of a meal plan, as an invariable and considered basic and fundamental therapeutic method for the psychiatric approach to anorexia nervosa. This plan is prescribed to the *patients* that must follow it scrupulously – they can’t change the amounts or the aliments prescribed. The foundation of this rigidness is that the weight gain must be gradual and, fundamentally, controlled (By the physician? By the patient?). Hence, patients aren’t allowed to eat, for example, chocolates, cakes or very highly caloric foods. The argument is that, if they eat those aliments, they might not eat those that are essential to the good functioning of the organism, due to the compensation logic characteristic of the disorder – that is, if the patient eats a chocolate, skips dinner that day and possibly lunch on the next, to compensate the very high caloric intake of chocolate.

The question here is trying to understand if the medical intervention, this way conceptualized and practiced, isn’t strengthening anorexia, instead of weakening it. If anorexia is a disorder of the control domain, the meal plan (as well as the pharmacological control of the emotions) seems to meet the proper logic of the disorder – that is, the control of the ingestion of food (to the minimum gram), the conception that there are, in fact, forbidden (“poisoned”) aliments, the self-control and inhibition of spontaneity and pleasure. If anorexia already imposes an interdiction to pleasure, the medical approach seems to legitimate this interdiction, forbidding and punishing (at least, tacitly) the spontaneous willingness to eat a pleasurable food.

As we could verify by the cases that we’ve followed, the commitment to the meal plan seemed to constitute, for all the patients, a maintenance factor of anorexia instead of a therapeutic means to an effective recovery.

Ana (19 years old; anorectic since 16), for example, was totally devoted to her psychiatric accompaniment and, as a good patient, completely obedient to her prescriptions. She assumed herself as an anorectic in the recovery process, although she was in the same stage of “evolution” since she had 16 years old (she had had several hospitalizations whenever the weight became a little lower than the usual very low). The meal plan turned out to be the strongest factor against her recovery. It was the meal plan that kept her anorectic for so long. She felt she was almost committing a crime whenever she ate more than it was prescribed, although her main wish was to gain weight, so that she could go back to school and have a normal life. She described her body as a skeleton and she wanted to recover the body curves she used to have almost four years before. Even so, she had a tremendous difficulty in allowing her body to grow, saying, many times, that her meal plan was very specific and that she couldn’t gain weight eating whatever she wanted but what her psychiatrist thought to be the best for her.

Maria (30 years old; anorectic since 13), followed meal plans since she was 17 years old. The slightest possibility of not obeying her plan was felt like a crime. She wasn’t able to follow the challenge (proposed by our intervention) of eating a very desired and pleasurable food. We should notice that she lived with anorexia since she was 13 and that she had had psychiatric accompaniment since 17. In all these years, she has never left anorexia, although she obeys orderly to her dietary plans prescribed by the psychiatrist. Maria hasn’t yet recovered from her anorexia yet.

Sofia (20 years old; anorectic since 11), followed meal plans since she was 12 years old and, once again, she has always lived with anorexia.

On the other hand, we should look at the cases of Sara (23 years old, anorectic since 17), Isabel (16 years old, anorectic since 14) or Luisa (26 years old, anorectic since 14). They have had full recovery of their anorexia, because they agreed to give away control – that is, they renounced their secure and comfortable meal plans, in order to a real recovery from anorexia, this, of course, in the context of a supportive therapeutic group intervention. What we’ve realized is that, the more committed the patient was to the psychiatric treatment, the more difficult it was to actually leave anorexia.

Additionally, as we’ve said earlier, the psychiatric accompaniment itself, oriented for weight control (i.e., for the numbers that matter for anorexia) seems to be agreeing with the disorder structure.

It’s in this sense that we formulate psychiatric intervention as a “pact with anorexia”, instead as with the *anorectic*: it makes a pact with the logic, the prohibitions and the self-impositions of the disorder, “telling” it – “You can stay in the patient, but just let her gain a minimum weight so that she can survive in this life”. Is this, really, the resolution of anorexia nervosa? It seems to us that, within this paradigm, anorexia is considered to be a chronic disease and that this kind of intervention empowers the disorder and not the woman. Well, as we preconize psychological intervention, it should always seek the empowerment of the person, of one’s capacity for multivocality of identity and for the adoption of a life narrative promoter of a greater well-being.

Manuela (56 years old; anorectic since 20), is a paradigmatic example of our theory. This woman, extremely emaciated, hardly moved and was so fragile that the simple fact of sitting on a chair was difficult and painful. She had been receiving psychiatric treatment since she was 25. She had never recovered. In fact, the “treatment” kept her anorectic for all those years: that is, counting the grams she ingested, controlling her weight and BMI weekly, totally unfamiliar to the feelings of freedom and pleasure, desautonomized and controlled – by herself and by the doctors. She alternated between ambulatory treatment and hospitalization several times a year, during all her adult life. This way, although her parents couldn´t take care of her anymore, she got substitutes: her psychiatrist and the medical staff of her second home – the hospital.

### 3.3 The Promotion of the “Anorectic Career”

The medical model of intervention seems to promote the ingress and progression in what we call the *anorectic career*, throughout which the woman adopts an *anorectic identity*, colonizer of her existence.

The routine of psychiatric sessions for weight control and establishment of meal plans, the *professionalizing* encounters in the waiting room with other anorectic patients (in which they teach each other strategies for losing weight, deceiving the doctors and those who control their weight and alimentation) and the hospitalizations (with the frequent regimes of rewards and punishments) initiate and arrest the patient to a variety of habits in which they become experienced and eximious.

For the “professional anorectics”, everything about anorexia is known and inspected, experimented and analyzed. The experience on anorexia is valued among anorectics in a scale of victimization and self-commiseration, where the “most ill” (i.e., the one with more hospitalizations, lowest weight obtained, higher death risk,…) goes first. It’s usual for the patients to talk about their “conquests”, as if they were professional achievements. This must be recognized and the victimization necessity signified for an effective therapeutic and autonomizing progression.

The patients seemed to be constructing their personal narratives anchored in the disorder. The obvious contamination of the patients’ discourse with the terms of the disorder (like, self-labeling themselves as “restrictive anorectics”) seem to hide the capacity for multivocality of the identity and restrict the construction of more adaptive meanings.

In fact, through the medical approach, the necessity of external (secure) parameterization and control of these women is carried to the extreme: the body is totally scrutinized, the weight and the BMI regularly controlled and the food ingestion totally calculated, determined and prescribed by the exigency and totalitarianism of the meal plans. In conclusion, through the medical model of intervention, anorexia is well nourished, for it goes stronger and confirmed.

## 4. Final Remarks: The Essential Underpinnings of an Intervention in Anorexia Nervosa

Given our conceptualization of anorexia nervosa and our critical perspective about the mainstream method of Psychiatric intervention, we would like to propose some fundamental pillars to an effective therapeutic intervention with anorectic patients.

### 4.1 Liberation

If anorexia is a disorder of control, the major necessity and the key-ingredient for these patients is liberation – liberation from the numbers of weight, BMI, calories; liberation from eating control patterns (meal plans), from weight checking, from body checking; liberation from the identity-label of anorexia.

The resolution of anorexia cannot be the control over food ingestion and overweight but exactly the opposite: these women should learn to eat whatever they like and whenever they want and to look at body weight as secondary to their well-being.

An intervention with these patients should challenge them to experiment liberation at all levels (in the context of a supportive therapy), including giving away hypervigilance. This seems to be much more effective when the body is included in the intervention, instead of using a strictly verbal psychotherapy. We must recognize that control, hypervigilance and liberation itself are lived in and through the body. Hence, using movement exercises (which implicate trusting the body to the group, for example) or relaxation exercises can be powerful tools in following the purpose of liberation.

### 4.2 Pleasure

As we’ve described above, these patients seem to have a commitment to unhappiness and victimization. Pleasure was (unconsciously) seen as morally wrong and, thus, it was interdicted to them at all levels (and not just at the level of food restriction, especially of those pleasurable foods that were the most forbidden ones, even for the doctors).

Probably, alexithymia (the word comes from Greek *lexis* – absence and *timia* – emotion, and indicates a difficulty in verbalizing and describing feelings, as well as bodily sensations – vd. Sifneos, 1973; Taylor &Doody, 1985) can be related to this intentional interdiction of pleasure.

According to Alexander Lowen (1970), a central mentor of Bioenergetic Analysis, pleasure corresponds to the perception of an expansive movement inside the body, a flow of feelings and energy towards the periphery of the body. It is, for that reason, uncontrollable. Suppressing the impulse, the desire and the excitation – as a way of self-defense – the person won´t feel anxiety, nor pain, but will be interdicted from pleasure, through the construction of an “armor” that, with time, according to Lowen, becomes chronic and unconscious. The person starts to live in a rigid way, imprisoned physically and emotionally within the armor that has been built to his own protection.

These women inhibit every single impulse (towards pleasure), as if pleasure could deviate them from total control that they fight exhaustively to exert, maintaining themselves imprisoned in their “armors” that seem (and only seem to turn them into rigid, cold, alexithymic and indifferent to life women.

Despite the immeasurable efforts for not feeling the necessity of pleasure, this, as Lowen (1970) explains, is a biological orientation. The essential goal of life, according to Freud’s theory or to António Damásio (vd. Damásio, 1994, 1999 & 2003), is pleasure and never pain. This way, the desire for pleasure emerges naturally in all human beings – and, of course, in our anorectic patients. In fact, the desire for pleasure betrays their control and commitment to unhappiness: at the same time they oppress themselves and restrict severely their alimentation, specifically in what concerns pleasurable and forbidden foods, they develop an obsession for everything that is related to food, recipes and culinary programs (Crisp, 1995; Girard, 2008; Sampaio et al., 1999). Food becomes the only thing they can think about – not just in terms of what they should ingest and their caloric values, but also in terms of absolutely forbidden aliments and elaborated recipes, compensating them, indirectly and vicariously, through the reading and preparation of elaborated recipes, watching culinary programs and by cooking (to others, of course), becoming eximious cooks, without tasting a bit of what they make.

It’s in this sense that we say that desire (uncontrollable force came from the enemy – the body) constitutes a betrayal to their decision of controlling everything, of not feeling, of stagnating, of not living.

### 4.3 Trusting the Body

As we’ve said previously, their bodies were treated and felt like enemies and, thus, treated with suspicion. Learning to trust in their own bodies and bodily sensations, developing the interoceptive awareness and sensitivity, and giving up the anorexia external criterions of self-worth were, therefore, central aspects of the intervention.

The major challenge overcome by the patients was to (return to) trust in their bodies’ capacity for finding a balance and to respect that balance, specific to each body, idiosyncratic and, therefore, non-determined by external criterions (like body weight, calories, body fat percentage or clothing size). This personal balance shouldn’t, thus, be subdued to the weight and BMI normalization factors.

This central pillar of the intervention includes, for example: respect the body when it is tired; eating normally throughout the day when it feels hungry; trusting the body’s ability to function in accordance to its own inborn intelligence; trusting the body’s ability to find its particular and unique balance.

Once again, the use of the body was a major help to this purpose. Patients were able to feel their bodies and learn to trust them in various ways, as well as to experiment, through movement, the innate tendency of the body towards equilibrium.

### 4.4 Promotion of Individuation and Adulthood

According to what we’ve explained, these women need to be encouraged to growup, to pursue an adult identity and to walk through the individuation process to find themselves as unique, separate human beings.

Within the context of our intervention with women diagnosed with anorexia, this main goal involved the following three fundamental tasks:

- Respect and promote their individuality and autonomy;
- Bring up to consciousness and to the body the dimensions of sexuality, sensuality and femininity;
- Encourage the formulation and persecution of life projects.

### 4.5 Accepting Uncontrollability as Prerogative of Life

Finally, these women need to learn how to accept uncontrollability in life (especially within the conjuncture of contemporary risk societies), the major factor they try so hard to fight. This only happens when self-confidence is established and they feel capable of receiving the world and life as they are: unpredictable, uncontrollable, erratic.

With a sense of liberation relative to their feelings, movements and actions, with the capacity for feeling pleasure without any guilt, with the corporal assurance and the confidence in one’s own body and with the capacity for assuming themselves as adult, responsible women, they are capable of accepting the uncontrollability of life, essentially because they feel themselves as trustworthy, truthful and authentic. In the line of attachment theory, we can comprehend that once the person has confidence and is capable of relying on someone (or on oneself) as his “safe harbor”, the person can explore the world, always knowing that, in spite of the frustrations that can come, they can always return to their harbor where they are reassured.

The use of the body, expressive movement and dance allowed us to work with central issues of the phenomenological experience of women with anorexia nervosa, in a much more direct way than an exclusively verbal psychotherapy would permit. We must recognize that the preferential vehicle of these women to express their inner suffering is, precisely, the body.

Their trunks, strangers to movement, and their bodies, strangers to touch, needed to be awakened and familiarized with the language of pleasure. Our intervention was able to challenge their rigidness, both postural/motoric and psychological, surpassing the concreteness within which they were prisoners, through the use of symbolic language. The body weight had to be felt and symbolized, as well as lightness had to be signified and deconstructed. These women had to learn how to trust themselves, giving away hypervigilance and expanding their kinespheres (the space surrounding the body) – that is the same to say, their space in the world or, rather, their world.

The group, in a body-oriented psychotherapeutic intervention, promoting a sense of confidence, empathy, safety and corporal assurance, helped these women to surpass some of the critical questions they face: How does it feel to lose control? What happens if I lose it? Who am I without anorexia?

## Footnotes

Anorexia nervosa occurs in females 10 times more than in males (vd.APA, 2000; Woodside, et al., 2001). About the fact that anorexia nervosa still remains, in its majority, a feminine phenomenon, it would be necessary an essay exclusively dedicated to this theme.
